# A Phase I Trial of Iopofosine I 131 and Dexamethasone in Patients with Relapsed/Refractory Multiple Myeloma

**DOI:** 10.3390/cancers18132044

**Published:** 2026-06-24

**Authors:** Sikander Ailawadhi, Jennifer L. Peterson, Kate Oliver, Jarrod Longcor, Natalie Callander

**Affiliations:** 1 Division of Hematology/Oncology, Mayo Clinic, Jacksonville, FL 32224, USA; 2 Department of Radiation Oncology, Mayo Clinic, Jacksonville, FL 32224, USA; 3 Cellectar Biosciences, Florham Park, NJ 07932, USA; 4 Division of Hematology/Oncology and Palliative Care, University of Wisconsin Carbone Cancer Center, Madison, WI 53706, USA

**Keywords:** myeloma, refractory, phospholipid ether, iopofosine

## Abstract

**Background/Objectives**: Patients with relapsed/refractory multiple myeloma (RRMM) have poor survival outcomes and limited treatment options. The increasing utilization of multiagent therapies for earlier lines of treatment and novel drug classes including BCMA-targeting agents in subsequent lines of therapy has created a need for innovative treatment platforms in patients who have relapsed after or are refractory to these current standard-of-care approaches. Iopofosine I 131 is a ^131^iodide (^131^I)–phospholipid conjugate that exploits the selective uptake and retention of phospholipid ethers through lipid rafts to facilitate tumor delivery of ^131^I. **Methods**: The objective of this phase 1 dose-escalation study was to assess the safety and tolerability of single and fractionated dose schedules of iopofosine I 131 + low-dose dexamethasone in heavily pretreated patients with RRMM. **Results**: The most common AEs overall were cytopenias, notably thrombocytopenia (93.5%), lymphopenia (74.2%), anemia (71%), leukopenia (61.3%), and neutropenia (58.1%). All patients experiencing hematologic adverse events recovered from those events with median time of recovery 21 days post-nadir. Nonhematologic adverse events were mostly limited to Grade 1 and 2. Dose-limiting toxicities included four Grade 4 thrombocytopenia events lasting longer than 7 days, one Grade 4 neutropenia, and one Grade 3 insomnia. The DMC determined 31.25 mCi/m^2^ was the maximum tolerated dose in the single-dose group, and 20 mCi/m^2^ × 2 doses was the maximum tolerated dose in the fractionated-dose group. For patients monitored through 85 days following the first infusion, 22 of 26 (84.6%) achieved stable disease after treatment, and 4 of 26 (15.4%) achieved a partial response. **Conclusions**: A favorable safety and tolerability profile and preliminary clinical activity support further development of iopofosine I 131 in RRMM.

## Simple Summary

Despite significant therapeutic advances, patients with multiple myeloma (MM) who no longer respond to available treatments often have very limited options and face poor outcomes. This study tested a new targeted radiopharmaceutical therapy called iopofosine I 131, given with low-dose dexamethasone, in people who had relapsed after many prior treatments. The primary goal of the study was to understand the safety and tolerability of different dose levels and schedules. The most frequent side effect was low blood count, which generally improved approximately three weeks after reaching their nadir, with all other side effects primarily low grade. A single maximum tolerated dose and a highest safe fractionated dose were identified. After treatment, most patients had their disease stabilize with a subset showing clear reduction in disease burden. These early results suggest that iopofosine I 131 could become a promising new option for difficult-to-treat relapsed and/or refractory MM.

## 1. Introduction

Multiple myeloma (MM) is the second most common hematologic malignancy representing approximately 18% of all hematologic cancers in the United States [1]. Over the past decade, novel treatment options have significantly improved overall outcomes for patients with MM [2]. The universal use of proteasome inhibitors, immunomodulatory drugs, monoclonal antibody agents, novel small molecule inhibitors [3,4], and increased use of stem cell transplantation and targeted cellular therapies (i.e., chimeric antigen receptor T-cell (CAR-T), bispecific antibodies) have improved survival [5,6,7,8]. For patients with relapsed or refractory MM, current guidelines recommend triplet therapy when feasible, incorporating agents with different mechanisms of action than prior lines of therapy [2]. However, achieving a cure remains elusive and relapses occur in most patients. Patients with multiple relapses or those who are multiple-class refractory have poor outcomes and short survival [2,9]. The movement of anti-CD38 monoclonal antibodies and CAR T-cell therapies into significantly earlier lines of therapy has resulted in many patients becoming triple-class exposed or refractory by their second or third line of treatment [2]. The increasing use of triplet and quadruplet drug combinations has prolonged the duration of response but patients ultimately relapse and can be refractory to agents previously used [2,4,10,11,12], highlighting a need for the development of new treatment platforms.

Targeted radiopharmaceutical therapies have emerged as an important therapeutic modality in oncology by enabling the selective delivery of cytotoxic radiation to tumor cells while limiting exposure to normal tissues. Unlike conventional external beam radiotherapy, targeted radiotherapies exploit tumor-associated biologic features, including receptor expression, metabolic activity, or cell membrane composition, to preferentially localize radioactive payloads within malignant tissue. Recent clinical successes in both solid tumors and hematologic malignancies have demonstrated the potential of targeted radiotherapy to produce meaningful and durable antitumor responses, including in heavily pretreated patient populations. Lutetium-177 PSMA-617 (^177^Lu-PSMA-617) demonstrated significant improvements in progression-free and overall survival in metastatic castration-resistant prostate cancer in the phase 3 VISION trial, leading to its FDA approval in 2022 [13]. Similarly, ^177^Lu-DOTATATE has shown efficacy in somatostatin receptor-positive gastroenteropancreatic neuroendocrine tumors, with the NETTER-1 trial demonstrating marked improvement in progression-free survival compared with high-dose octreotide [14]. Furthermore, advances in radionuclide selection, dosimetry, molecular imaging, and theranostic approaches have accelerated the development of personalized treatment strategies designed to optimize the therapeutic index of these agents. These clinical successes have underscored the broader applicability of targeted radiopharmaceutical therapies across hematologic and solid tumor malignancies and support the continued investigation of targeted radiopharmaceutical approaches, such as iopofosine I 131, in MM.

Iopofosine I 131 (iopofosine, formerly referred to as CLR 131) is a ^131^iodide (^131^I)–phospholipid conjugate that exploits the selective uptake and retention of phospholipid ethers through lipid rafts to facilitate tumor delivery of ^131^I [15]. Lipid rafts, which play important roles in cancer cell proliferation, survival, and metastasis, are markedly overexpressed by malignant cells compared with normal cells, constituting a promising mechanism for cancer-targeted therapies [16,17,18]. In preclinical models, iopofosine has been shown to rapidly accumulate in tumors, leading to delayed tumor growth, reduced tumor volume, and prolonged survival in both solid and hematologic cancer models, including MM [19,20]. Furthermore, in preclinical models, fractionated dosing of iopofosine was better tolerated than an equivalent single dose of the drug [19].

Herein, we report the results of a phase 1 dose-escalation study evaluating both single and fractionated dose schedules of iopofosine in heavily pretreated patients with RRMM. A fractionated dosing strategy was utilized as a potential way to increase the total administered dose with minimal impact on toxicity [19,21].

## 2. Materials and Methods

This phase 1, open-label, multi-institutional, sequential dose-escalation study evaluated the safety and tolerability of a single or fractionated dose of iopofosine (infused intravenously (IV) over ~30 min) in combination with weekly dexamethasone (DEX; 40 mg/week for 12 weeks) administered to heavily pretreated RRMM patients (NCT02278315). The primary objective was to determine the safety and tolerability of iopofosine in such patients. The secondary objectives were to evaluate early signs of therapeutic activity and to gain insight into potential phase 2 dosing considerations for iopofosine in this patient population. The study protocol complied with the ethical standards outlined in the Helsinki Declaration of 1975 (as revised in 1983). It was reviewed and approved by the Institutional Review Board and Radiation Safety Committee at each study site. Informed consent was obtained from all participants involved in the study. Iopofosine was produced by Center for Probe Development and Commercialization (Hamilton, ON, Canada).

### 2.1. Study Participants

Participants who had relapsed after, or were refractory to, a proteasome inhibitor (PI) or immune modulator (IMiD) containing antimyeloma regimens with histologically confirmed MM (bone marrow biopsy within 28 days of study drug infusion) and measurable disease as per the IMWG criteria were eligible [22,23]. Inclusion criteria included evidence of progressive disease [22] and Eastern Cooperative Oncology Group (ECOG) performance status (PS) of 0 to 2. There was no upper limit to the number of previous therapies a patient could have received. Patients with non-secretory disease were allowed on a case-by-case basis.

To be eligible for this trial, study participants had to have baseline laboratory values consistent with adequate liver (alanine aminotransferase < 3× the upper limit of normal), renal (estimated glomerular filtration rate > 30 mL/min/1.73 m^2^), and bone marrow function (white blood cell [WBC] count > 3000/mL; absolute neutrophil count > 1500/mL, platelet count > 100,000/μL without full-dose anticoagulation or >150,000/μL with full-dose anticoagulation therapy, and hemoglobin > 8 g/dL). Patients who were being treated with anticoagulants at the time of enrollment were allowed to continue, provided the anticoagulant they were using was reversible and that reversal would not be life-threatening.

Patients were excluded if they had ongoing toxicity (Grade ≥ 2) from prior antimyeloma therapy, had a history of hypersensitivity to iodine, had received prior external beam radiation during which >20% of total bone marrow received more than 20 Gy, had received prior radioisotope therapy, or had received prior whole-body or hemi-body radiation treatment.

### 2.2. Study Design

Participants were sequentially assigned to a single- or fractionated-dose cohort (Table 1). Each cohort was planned to enroll at least 3 patients in a standard 3 + 3 design. Over-enrollment was allowed in cohorts to ensure a minimum of three evaluable patients in each cohort, accounting for any participants who may discontinue early. All individuals who received a dose of iopofosine were included in the safety assessment dataset. The efficacy data set included participants who continued to day 64 post-infusion. Participants who discontinued before completing day-64 procedures could be replaced within each treatment cohort. Patients were followed up for a minimum of one year or until death. The extended safety and efficacy evaluation period of 64 days was based upon the 8-day half-life of iodine 131, allowing adequate time for observation of adverse events and assessment of response.

**Table 1:** Dose assignment and disposition.

	Dose Cohort (mCi/m^2^)							
	12.5 × 1 (A)	18.75 × 1 (B)	25 × 1 (C)	31.25 × 1 (D)	15.63 × 2 (E) *^,†^	18.75 × 2 (F) *	20 × 2 (G) *	Overall
Treated, n	5	5 ^‡^	4 ^§^	4	4	5	4	31
1 dose	5	5	4	4	-	1 ^	1	19
2 doses	-	-	-	-	4	4	4	12
Safety analysis, n	5	5	4	4	4	5	4	31
Efficacy analysis, n	4	4	4	3	4	4	3	26
Baseline BSA (m^2^), mean ± SD	2.00 ± 0.28	1.86 ± 0.32	1.98 ± 0.23	1.74 ± 0.18	2.06 ± 0.23	1.76 ± 0.19	2.01 ± 0.23	1.91 ± 0.25
Total iopofosine dose (mCi), mean ± SD	24.5 ± 4.1	35.5 ± 6.2	51.2 ± 7.2	55.3 ± 5.9	65.2 ± 9.6	62.8 ± 19.9	84.5 ± 10.9	52.4 ± 19.1
Total DEX dose (mg), mean ± SD	320.8 ± 167.3	400.0 ± 123.3	460.0 ± 40.4	430.0 ± 100.0	290.0 ± 179.9	256.0 ± 180.8	290.0 ± 66.3	347.2 ± 142.8
DEX duration (day), mean ± SD	66.4 ± 30.8	71.2 ± 21.7	84.5 ± 1.0	76.0 ± 17.3	80.8 ± 6.0	59.8 ± 34.1	70.3 ± 13.8	72.0 ± 21.5

Abbreviations: BSA = body surface area; DEX = dexamethasone; SD = standard deviation. * Fractionated doses were administered IV on days 1 and 7 (±1 day). ^†^ The same target total dose as cohort D. Iopofosine administered was higher due to higher BSA in this cohort (Table 2). ^‡^ One patient was enrolled with 4% plasma cells on screening biopsy but clear evidence of disease progression. ^§^ At the time of screening, 1 patient had isolated lymphopenia (with no other cytopenias) that was considered unrelated to prior therapy. ^ After the first dose, the patient discontinued from the study and died on day 8, unrelated to study medication.

**Table 2:** Baseline demographics at screening for 31 enrolled patients.

	Dose Cohort (mCi/m^2^)							
	A (n = 5)	B (n = 5)	C (n = 4)	D (n = 4)	E (n = 4)	F (n = 5)	G (n = 4)	Overall (n = 31)
Age (y), mean ± SD	68.4 ± 8.3	69 ± 11.7	71 ± 7.7	61.5 ± 9	71 ± 4.1	72.8 ± 9.2	71.3 ± 7.8	69.4 ± 8.5
Men, %	80	40	50	50	75	40	75	58.1
Median lines of systemic prior therapy	5	4	6	6	4	3	4	4
Prior radiation treatment, %	40	0	50	50	25	0	75	32.3
Cytogenetics: high risk, %	0	20	25	0	50	40	25	23
Median bone marrow involvement, %	25	40	10	25	10	25	5	18
Renal impairment (eGFR < 40 mL/min), n	0	20	0	25	0	20	0	9.7
Previous treatments, % of study participants *								
Alkylating agent	60	80	100	75	75	80	75	67.7
Transplant	40	80	100	75 ^†^	25	40	50 ^†^	58.1
Monoclonal antibody	20	20	25	75	25	80	50	41.9
Non-alkylating agent ^	10	0	25	0	0	0	25	9.7
IMiD exposure	100	100	100	100	100	100	75	96.8
IMiD refractory	80	100	75	100	75	80	50	81
Proteasome inhibitor exposure	100	100	100	100	100	100	100	100
Proteasome inhibitor refractory	60	60	50	100	75	80	50	68

Abbreviations: eGFR = estimated glomerular filtration rate; IMiD = immunomodulatory agent; MM = multiple myeloma; SD = standard deviation. * All patients were previously treated with proteosome inhibitors, immune modulators, and corticosteroids. Additional prior treatments are shown here. ^ Non-alkylating agents include, but are not limited to: investigational therapies, etoposide, doxorubicin. ^†^ One patient received 2 previous transplants.

No additional MM-directed therapies, including external beam radiation, were allowed during the treatment phase. At the discretion of the study investigators, hematologic toxicities were managed with platelet or red blood cell transfusions, or filgrastim until bone marrow recovery. Nonhematologic toxicities were managed with supportive care. As there are no known drug interactions with iopofosine, concomitant medications for conditions unrelated to MM were allowed at the discretion of the study investigator.

Data from each dose level was reviewed by the data-monitoring committee (DMC) before allowing enrollment into the next higher-dose cohort.

### 2.3. Iopofosine Administration

**Figure 1:**
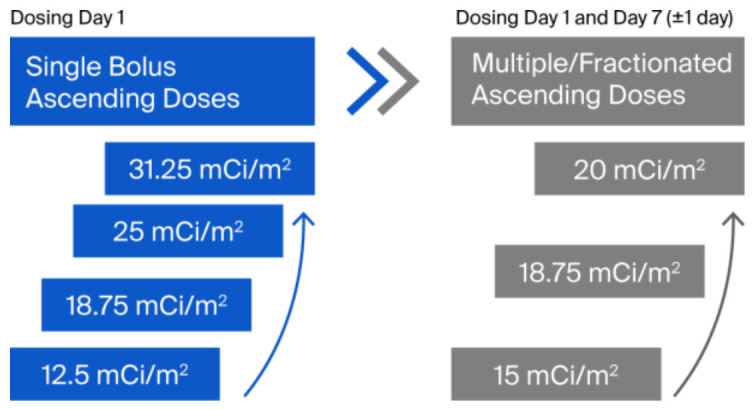
Study schema for phase 1 trial of iopofosine I 131 in RRMM. For participants receiving a single dose of iopofosine, administration occurred on day 1. Fractionated doses of iopofosine were administered on day 1 and day 7 (±1 d). All doses were calculated using the participant’s body surface area (BSA; Mosteller formula), not to exceed a BSA of 2.5 m^2^.

Ready-to-infuse preparations of iopofosine (18-(p-[^131^I]iodophenyl)octadecyl phosphocholine in sodium chloride injection with ethanol, polysorbate 20 National Formulary, and sodium ascorbate) were provided to study sites in single-dose vials and stored in lead-lined containers until use. Based on an 8-day half-life for ^131^I, sufficient material was provided in each vial to achieve the assigned dose on the scheduled infusion day. Pretreatment dosimetry studies were not required at the study site. Infusions could be administered in an in- or outpatient setting per study site workflow. On the day of administration, iopofosine was diluted, filtered through a 0.2 µm filter, and infused through a free-running peripheral IV catheter over approximately 30 min. Table 1 shows the administered dose of radioisotope for each dose cohort. Patients were clinically monitored during and up to 4 h after infusion for infusion-related reactions and other adverse events. During this time, participants also completed serial 12-lead electrocardiograms for assessment of cardiac safety. A single cycle of iopofosine was administered as shown in Figure 1.

Study participants received thyroid-protection therapy (potassium iodine 130 mg once daily or similar) starting 1 day before iopofosine infusion and continuing for 14 days (patients receiving a single dose of iopofosine) or 22 days (patients treated with fractionated doses), in accordance with standard guidelines [24]. Participants received guidance on ways to lessen the risk for ^131^I exposure for 1 to 3 weeks after infusion [24].

Low-dose DEX (40 mg/week for 12 weeks with the option to continue beyond the 12 weeks at the study investigator’s discretion) was initiated on the day of iopofosine infusion. The dose of DEX could be reduced as needed for tolerability as per physician preference (Table 1).

Low-dose dexamethasone was included in the treatment regimen to provide background disease control, reduce disease-related inflammatory symptoms, and reflect contemporary clinical practice in myeloma management, while minimizing the potential for confounding efficacy and safety assessments of iopofosine. Corticosteroids, including dexamethasone, are well established as a foundational component of multiple myeloma therapy due to their direct antimyeloma activity and ability to improve tolerability of treatment regimens through reduction in cytokine-mediated symptoms, infusion-related reactions, pain, fatigue, and constitutional symptoms commonly observed in heavily pretreated patients.

In the relapsed/refractory setting, low-dose dexamethasone is frequently utilized as a backbone therapy in combination with novel agents because it can provide modest antimyeloma activity without introducing substantial additional toxicity or myelosuppression. This is particularly relevant in early-phase radiopharmaceutical studies, where preservation of bone marrow reserve and avoidance of overlapping hematologic toxicities are critical considerations. The use of low-dose dexamethasone therefore supports patient stability and treatment tolerability while allowing clearer assessment of the safety, dosimetry, pharmacokinetics, and preliminary antitumor activity attributable to the investigational targeted radiotherapy.

Additionally, inclusion of dexamethasone aligns with established treatment paradigms in relapsed/refractory multiple myeloma and enhances the clinical interpretability and future translatability of study results. The selected low-dose regimen was intended to minimize the likelihood that observed responses would be driven primarily by corticosteroid exposure while still providing supportive antimyeloma benefit and symptomatic management in this heavily pretreated patient population.

### 2.4. Assessments

Patients were assessed for hematologic and chemistry analyses before dosing and weekly after iopofosine infusion until day 85. The severity of adverse events (AEs) was determined using the Common Terminology Criteria for Adverse Events v4.03. Serious AEs included those considered life-threatening, requiring hospitalization, or resulting in a persistent incapacity or death. Dose-limiting toxicity (DLT) was defined as toxicities not clearly related to disease progression or intercurrent illness and included Grade 3 or higher febrile neutropenia or any Grade 4 hematologic toxicity (except for neutropenia without concurrent fever; thrombocytopenia present for less than 7 days without active bleeding; or lymphopenia), any nonhematologic Grade 4 toxicity, or any Grade 3 toxicity despite adequate supportive care (except for Grade 3 changes in renal function or clinically asymptomatic, correctable Grade 3 lab abnormalities). Serum thyroid-stimulating hormone and free T4 levels were determined at screening and on day 85. Coagulation function was assessed at screening. Response assessment was done by serum and urine tests as per International Myeloma Working Group (IMWG) [22] criteria at screening, days 22, 43, 64, and 85, or sooner at the principal investigator’s discretion [25].

### 2.5. Statistical Analysis

Descriptive statistics and analyses were provided by cohort and total administered dose group. Only observed data was used in the summaries and analyses; missing data were not imputed or carried forward. Continuous variables were expressed as the mean or median for patients in the overall population and each dose cohort. The efficacy analysis group included those participants who completed the day-64 assessment after iopofosine infusion. Secondary endpoints included overall response rate (ORR), clinical benefit rate (CBR), complete response (CR), very good partial response (VGPR), partial response (PR), progression-free survival (PFS), and duration of response (DoR).

## 3. Results

### 3.1. Baseline Characteristics

Between April 2015 and January 2020, 32 individuals were screened and 31 met the eligibility criteria for inclusion in the study. For the 31 treated participants, the median age was 69 years (range, 50–85 years); 58% were males. Median prior lines of systemic therapy in this cohort were four (range 2–12). Prior treatment characteristics for the different patient cohorts (cohorts A to G) by dose level and frequency are summarized in Table 2. Based on fluorescence in situ hybridization (FISH) and cytogenetic analysis, 23% were identified as high risk [26]. Renal insufficiency (eGFR < 40 mL/min) was seen in 6.4% and renal failure (eGFR ≤ 15 mL/min) in 3.2% of the patients. Additional baseline characteristics were similar across dose cohorts. At the time of enrollment, 8 of 31 were triple-class refractory. Overall, 58.1% of patients received a previous autologous stem cell transplant (ASCT).

Twenty-six patients (81.3% of enrolled participants) were considered evaluable for inclusion in the efficacy analysis as they received iopofosine and completed the day-64 post-treatment assessment. Five participants were excluded from the efficacy analysis: three due to disease progression before day 64, one was removed per investigator discretion, and one withdrew to transition to hospice care.

The first 18 patients were treated with a single dose (from 12.5 to 31.25 mCi/m^2^, cohorts A–D), and the next 13 patients were infused with fractionated doses (equal mCi/m^2^ doses ranging from 15.63 to 20 mCi/m^2^ administered 7 days apart; cohorts E–G; Table 1). All participants in the single-infusion cohort and 92% in the fractionated-dose cohort received their assigned dose (Table 1). Based on safety assessments, the DMC determined that cohort D (31.25 mCi/m^2^) was the maximum tolerated single-dose in this study and initiated the fractionated-dose cohorts, cohorts E–G). Cohort G (20 mCi/m^2^ × 2 doses) was the highest dose evaluated in the fractionated-dose group despite no fractionated-dose cohort achieving the 33.3% DLT criteria to establish the maximum tolerated dose. Of 31 treated study participants, five discontinued due to disease progression (16.7%); no patients discontinued due to treatment-emergent AEs. The median follow-up for participants was 15.6 months (range 0.2–65.6), with 10 patients (32%) alive at the time of data cut-off.

Although no DLTs were observed, dose escalation of iopofosine I 131 was discontinued after cohort G to limit total exposure and reduce the risk of cumulative bone marrow toxicity. Because the principal toxicity was hematologic, the DMC felt further escalation could have increased the likelihood of prolonged myelosuppression and potential marrow injury without materially improving the benefit–risk profile and recommended stopping the escalation at cohort G.

### 3.2. Safety Assessment

The most common AEs overall were cytopenias, notably thrombocytopenia (93.5%), lymphopenia (74.2%), anemia (71%), leukopenia (61.3%), and neutropenia (58.1%). Additional nonhematologic AEs included fatigue (64.5%), nausea (41.9%), dyspnea (32.2%), diarrhea (29.0%), hyponatremia, insomnia (25.8% each), QT prolongation on electrocardiogram, decreased appetite, hyperglycemia, and hypophosphatemia (22.6% each). Nonhematologic AEs were generally mild (Grade 1 or 2), transient, and managed with supportive care. Overall, the median time to nadir for hematologic events was 34 days after the initial dose, regardless of the dose. The median time to recovery to Grade 2 or better from cytopenias was 21 days post-nadir. The median time to recovery to Grade 2 or better was 5.5 days for hemoglobin (95% confidence interval (CI), 9–29), 9.0 days for absolute neutrophil count (ANC) (95% CI, 7–22), 24 days for WBC count (95% CI, 9–29), 36 days for platelet count (95% CI, 23–50), and 64 days for lymphocyte count (95% CI, 35–73). Nine participants in the single-dose cohorts received a median of five platelet transfusions (range 1–6) while 10 participants in the fractionated-dose cohorts received a median of four platelet transfusions (range 1–16). Two participants in the single-dose cohorts received colony-stimulating factors and three patients in the fractionated-dose cohorts received colony-stimulating factors. Ten participants in the single-dose cohorts received a median of two red blood cell transfusions (range 1–5) while seven participants in the fractionated-dose cohorts received a median of five red blood cell transfusions (range 1–7). There were no reported incidences of myelodysplastic syndrome in any participants who received iopofosine.

All participants experienced at least one Grade 3 or higher AE, the worst being Grade 3 in 19.4% of patients, or Grade 4 in 77.4%. Treatment-emergent AEs (TEAE) ≥ Grade 3 observed in more than 5% of patients are reported in Table 3. Treatment-emergent serious AEs (SAEs) were reported in 41.9% of participants. There were no clinically significant changes in liver or renal function or electrocardiogram results after any dose of iopofosine. Thyroid hormone levels and coagulation times were not altered by iopofosine infusion.

Potential DLTs (reviewed by the DMC) occurred in six participants (19.4%). These included one DLT of thrombocytopenia in cohort C (single dose) and one each of neutropenia and thrombocytopenia in cohort D (the maximum tolerated single-infusion dose group). In the fractionated-dose cohorts, one DLT of thrombocytopenia was noted in cohorts E and F while cohort G had one DLT of insomnia. The most common DLT was Grade 4 thrombocytopenia lasting longer than 7 days occurring in four patients. The onset of thrombocytopenia was on days 34 and 38 after a single infusion in cohorts C and D, and days 22 and 23 following the second infusion in cohorts E and F. The median duration of thrombocytopenia DLTs across all cohorts was 12.5 days (range 12–36). All were treated with platelet transfusions.

There was one death in cohort B attributed to rapid disease progression. After a single infusion, Grade 3/4 cytopenias developed as early as day 14 and it was determined this was secondary to rapid disease progression. This patient was withdrawn from the study on day 41 and started on salvage VDT-PACE treatment [27]. The patient died on day 56 after receiving iopofosine and after hospitalization for pneumonia and respiratory failure. There was one death in cohort F that occurred on day 8 after infusion of the first of two planned fractionated doses. The patient experienced tumor lysis syndrome 2 days after the first infusion, with septic shock and respiratory failure on day 8. This patient did not experience cytopenias before the onset of septic shock. The individual withdrew from the study on day 7. The death was not attributed to the study drug; the investigator considered underlying disease, spontaneous tumor lysis, and recent nephrostomy tube insertion as alternative etiologies for the septic shock and compensation for metabolic acidosis or pulmonary embolism as possible alternative etiologies for respiratory failure.

Based upon preclinical data and prior work with ^131^I, the total administered dose of 60 mCi was predicted to be where initial activity would be demonstrated and therefore an additional analysis was undertaken to evaluate the overall safety of patients who received <60 mCi total dose (n = 20), and those who received ≥60 mCi (n = 11). The majority of individuals in the ≥60 mCi group were treated with fractionated doses of iopofosine. Baseline demographics were similar between these two groups. A higher proportion of patients in the ≥60 mCi group underwent DEX dose reduction compared with patients in the <60 mCi group (63.5 vs. 15%).

**Figure 2:**
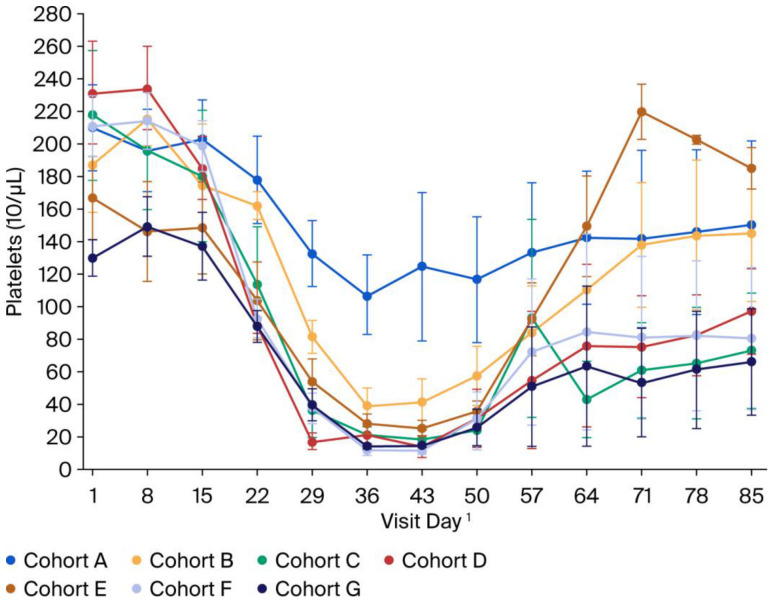
Platelet count following iopofosine administration. Change over time in platelet count (10/μL) after iopofosine infusion for all patients who received iopofosine. Figure shows mean ± SD at each time point for patients by assigned cohort. ^1^ Baseline (visit day 1) is defined as the last non-missing evaluation prior to iopofosine infusion.

**Figure 3:**
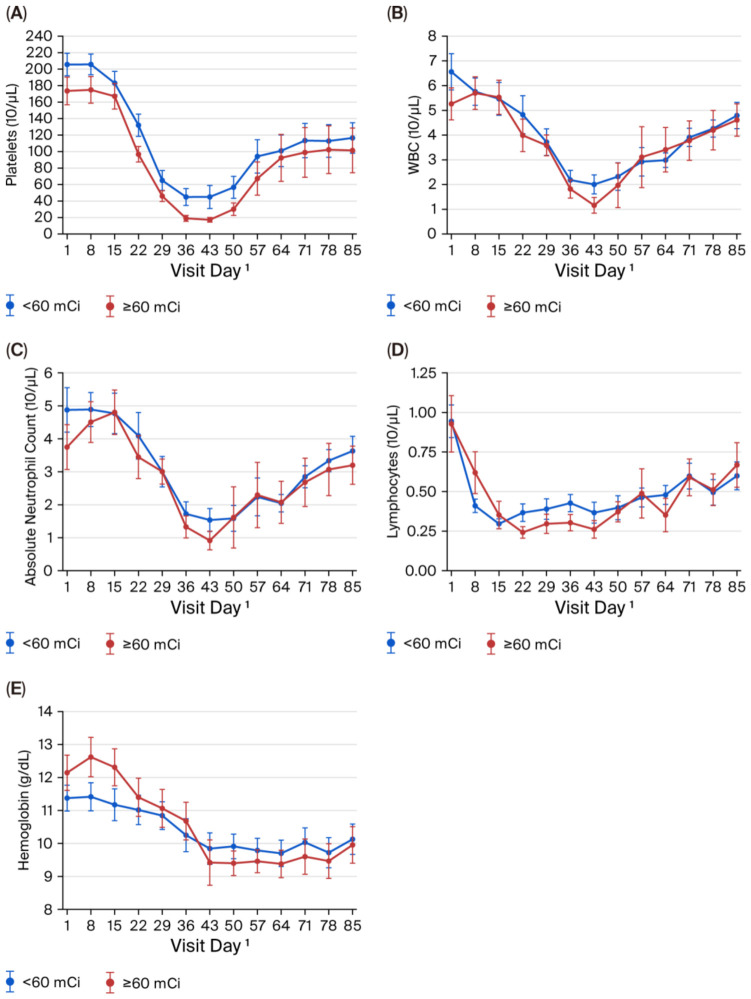
Hematologic toxicity following iopofosine administration. Change over time in hematologic parameters after iopofosine infusion. (**A**) Change in platelet count. (**B**) Change in white blood cell (WBC) count. (**C**) Change in absolute neutrophil count. (**D**) Change in lymphocyte count. (**E**) Change in hemoglobin. Figure shows mean ± SD at each time point for patients in the <60 mCi group (blue line) and ≥60 mCi (red line).

Overall, several hematologic and nonhematologic AEs were more frequent in the <60 mCi group compared with the ≥60 mCi group, including lymphopenia (95% vs. 36.4%, respectively), leukopenia (80% vs. 27.3%), neutropenia (65% vs. 45.5%), hyponatremia (35% vs. 9.1%), diarrhea (35% vs. 18.2%), and hypoalbuminemia (20% vs. 0%). The rate of thrombocytopenia was similar between the two groups and was consistent regardless of dose level (Figure 2). Time to nadir and time to recovery from nadir were similar for participants who received <60 and ≥60 mCi (Figure 3). The time to nadir and then to recovery was also similar between participants treated with <60 and ≥60 mCi (Table 4).

AEs that occurred more frequently in the ≥60 mCi group included fatigue (100% vs. 45%), dyspnea (45.5% vs. 25%), decreased appetite (36.4% vs. 15%), and insomnia (36.4% vs. 20%). More patients experienced serious AEs in the ≥60 mCi group than those in the <60 mCi group (54.4% vs. 35%).

### 3.3. Disease Response

**Figure 4:**
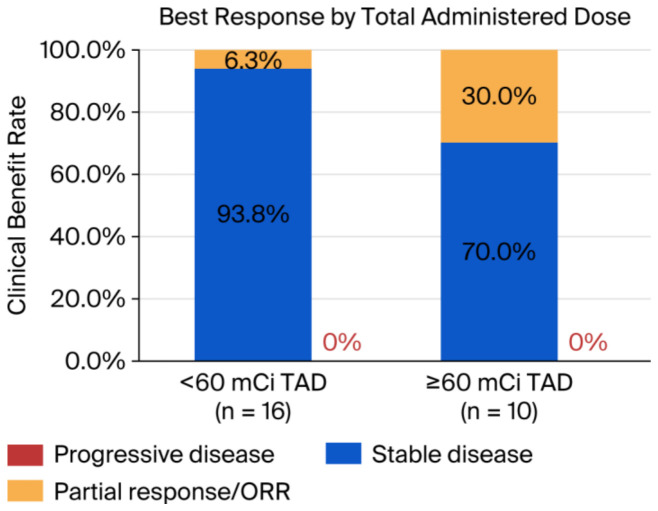
Best response by total administered dose.

**Figure 5:**
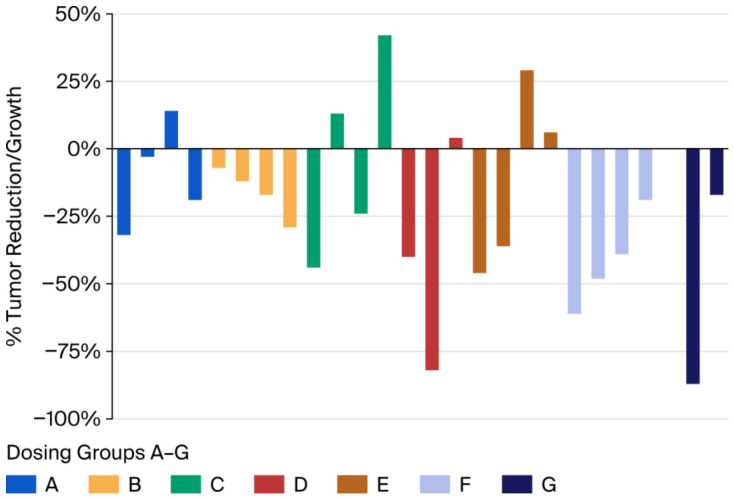
Responses following iopofosine administration. The best percent change in disease marker by disease cohort following iopofosine administration. Per IMWG criteria, a reduction ≥ 50% qualifies as a partial response.

Disease response to iopofosine showed an ORR of 15.4% (4 of 26 evaluable participants). Four participants achieved a PR and all study participants achieved stable disease (SD) or better responses for a CBR of 100% (Figure 4 and Figure 5). Of those achieving PR, two were triple-class refractory, 1 was penta-drug refractory. ORR was 6.3% among the patients who received <60 mCi total administered dose and 30% among those who received ≥60 mCi total administered dose. The ability of study participants to achieve ORR did not appear to be related to baseline ECOG PS, cytogenetic risk or International Staging System disease stage.

Median PFS was 101 days (95% CI, 79 to not estimable; NE) in the efficacy cohort (n = 26). When estimated by total administered dose of iopofosine, median PFS was 98 days (95% CI, 78 to NE) in the <60 mCi cohort, and NE for the ≥60 mCi cohort. Among participants with documented disease response, median DOR was 47 days (95% CI, 22–246) in the overall population (n = 26), 31 days (95% CI, NE) in the <60 mCi dose group (n = 16), and 63 days (95% CI, 22–246) in the ≥60 mCi dose group (n = 10).

## 4. Discussion

This study aimed to determine the safety and tolerability of iopofosine plus low-dose DEX in patients with heavily pretreated RRMM and to explore the recommended dose and schedule for a future phase 2 study. In a recent report, iopofosine demonstrated promising antimyeloma activity in patients with triple-class refractory MM and were relapsed from, or refractory to, anti-B-cell maturation antigen immunotherapy [28]. Fractionation of the doses was done to exploit the differential response of tumor and normal tissues to radiation. This approach allows for increased total dose delivery, promotes reoxygenation of hypoxic tumor regions, and enables redistribution of tumor cells into more radiosensitive cell cycle phases between fractions, ultimately maximizing therapeutic efficacy while minimizing late toxicity in surrounding normal tissues [29,30,31]. Available dosimetry data from prior studies demonstrated consistent biodistribution, target uptake, and organ retention across patients, with limited evidence of clinically meaningful interpatient variability in radiopharmaceutical localization or clearance kinetics. Given this reproducibility, total administered activity was considered an appropriate surrogate for tissue-absorbed dose, which is the primary determinant of both therapeutic efficacy and normal tissue toxicity in targeted radiopharmaceutical therapy. Under these conditions, increases in administered activity are expected to result in proportionate increases in absorbed radiation dose at the tumor and tissue level, thereby supporting the use of administered dose as a practical and biologically relevant exposure metric. Based on these observations, a target cumulative administered activity threshold associated with meaningful biologic and antitumor activity was explored.

The analysis of <60 mCi versus ≥60 mCi was a post hoc analysis conducted to further evaluate the relationship between total administered activity and clinical outcomes in the context of targeted radiopharmaceutical therapy. While tissue-absorbed dose is recognized as the primary determinant of both therapeutic efficacy and normal tissue toxicity, available dosimetry data from previous studies demonstrated consistent biodistribution and uptake characteristics across patients, with no evidence of substantial interpatient variability in target localization or organ retention. Under these conditions, total administered dose serves as a reasonable surrogate for tissue-absorbed dose, as increases in administered activity are expected to produce proportionate increases in radiation delivery at the tissue level. Accordingly, evaluation of outcomes as a function of total administered dose was considered scientifically appropriate and biologically relevant within this dataset. The analysis was therefore undertaken to assess potential dose–response relationships while leveraging the observed consistency in radiopharmaceutical uptake and dosimetric behavior across the treated population.

Overall, iopofosine treatment with single or fractionated doses was well tolerated. No infusion-site reactions were noted, and no infusions were discontinued due to AEs. No new safety signals were observed in this study compared with previously reported data on iopofosine [28,32,33,34,35]. Unlike traditional chemotherapy or biologic intervention trials, in which treatment is continuous, and response is typically evaluated after a set number of treatment cycles [36], iopofosine was of a fixed duration with treatment responses assessed after a single cycle.

Some degree of AEs were seen in all study participants, with 13 experiencing a serious AE (42%), and six experiencing a DLT (19%). Consistent with a population that has received multiple therapies directed to the bone marrow, marrow sensitivity to radiotherapeutics [37], and the known mechanism of action (MoA) for iopofosine [15,35], the most common AEs were predictable and transient hematologic events. Unlike other clinical studies with iodine 131 in hematologic malignancies, there was no maximum level of bone marrow involvement allowed in the study participants (range from 1 to 90-grade thrombocytopenia and lymphopenia were encountered in this study population more frequently than neutropenia or anemia (Table 3)). Platelet, neutrophil, WBC, and lymphocyte count nadirs were encountered between 15 and 43 days post-infusion, whereas hemoglobin levels declined gradually over time. Of interest, there was no correlation between the total dose of iopofosine administered (<60 vs. ≥60 mCi) and the degree or timing of cytopenias. Because of the phase 1 nature of this study and concerns that iopofosine could be associated with prolonged cytopenias, pretreatment hematologic parameters were set to be higher than what is typically required. Further evaluation with a larger population will be needed to elucidate the relationship between baseline bone marrow cellularity, disease burden and observed cytopenias.

Nonhematologic AE occurrence was remarkably low in the study, and these AEs were generally mild (Grade 1–2). Iopofosine did not have any effect on renal or liver function. Patients who developed infrequent Grade 3 or higher nonhematologic AEs were managed medically without any clinically significant sequelae. Some of these nonhematologic AEs may be attributed to DEX (e.g., insomnia) or thyroid protection used with iopofosine and not necessarily the radiopharmaceutical. The absence of new-onset peripheral neuropathy, peripheral edema, deep vein thrombosis, cardiotoxicity, embolism, or gastrointestinal toxicities attributable to iopofosine is encouraging compared with other targeted regimens used to treat RRMM and opens the possibility of combination treatments in future trials [2,4,10]. Considering the older age of patients with RRMM and associated poor prognosis related to renal insufficiency and other comorbidities [11,12], the absence of marked changes in renal function or liver enzymes and limited impact on PS after a single cycle of iopofosine further support continued development of this novel therapeutic approach for RRMM.

Although exploratory, the higher total administered dose achieved with fractionated dosing appears to be associated with improved disease response and without a notable increase in AEs. Although all participants achieved SD after treatment, PRs were noted in the highest single-dose cohort (group D) and fractionated-dose cohorts F and G. The ORR of 15.4% in the overall population and 30% in the ≥60 mCi cohort, and a CBR of 100% observed in this single agent, single-cycle trial compares favorably to some other phase 2 results of recent antimyeloma therapeutics including Selinexor + DEX [38] or belantamab mafodotin [39]. The duration of PFS observed in this study, notably in the ≥60 mCi cohort, suggests that iopofosine + DEX may offer a clinically meaningful treatment option for patients with heavily pretreated RRMM. Since this was primarily a safety and tolerability study, there are limitations on conclusions to be drawn about efficacy of iopofosine + DEX in this population. Subsequent studies of iopofosine will be designed and conducted to determine the optimal dose, schedule, regimen and combination to achieve better outcomes for MM patients.

In the present study, whether low-dose DEX contributed to observed clinical responses is unclear. Previous studies with high-dose pulse DEX have shown limited efficacy in treatment-resistant MM [40], whereas the addition of DEX in early trials of Selinexor had a variable effect on outcomes [41]. Further studies are needed to evaluate any additive role of low-dose DEX on the clinical benefit of iopofosine seen in this patient population.

The study design necessitated a minimum three-month period per cohort, comprising an extended two-month observation period post-treatment per patient for safety and tolerability assessment, followed by an approximately one-month period for the DMC review and determination of subsequent steps. This protocol, combined with the limited number of participating clinical sites, significantly prolonged the enrollment phase and impacted recruitment rates. Concurrently, the regulatory approval of novel therapeutic agents for RRMM during this extended period provided patients with alternative treatment options outside of clinical trials. These factors collectively may have influenced the observed initial efficacy signals in the study population. The prolonged enrollment period likely resulted in the recruitment of participants with more advanced disease and heterogenous prior treatments, potentially altering the efficacy profile of the investigational treatment.

An analysis of total administered dose, clinical activity, and safety demonstrated that responses emerged at approximately 60 mCi. Since dosing was based on BSA and the mean patient BSA was estimated to be 1.9 m^2^, a fractionated schedule of 15 mCi/m^2^ administered on days 1 and 7 with concurrent weekly oral low-dose DEX 40 mg for up to 12 weeks is the recommended phase 2 dose and regimen in relapsed or refractory MM patients.

## 5. Conclusions

As a potential treatment option for RRMM, iopofosine is well differentiated from currently available options by its unique MoA, dosing schedule, predictable and manageable hematologic AE profile, and low nonhematologic AEs. The absence of an effect on liver or renal function suggests that iopofosine may offer a treatment option for patients who may be frail or have organ dysfunction, which can be seen frequently in advanced RRMM [42]. The recovery of cytopenias over time indicates that repeat dosing of iopofosine treatment may be possible [30], further extending the potential clinical benefit. Additional studies will be needed to understand the potential for combining iopofosine with other systemic agents for RRMM and its potential use in the peri-stem cell transplant setting.

Results from this phase 1 dose-escalation study offer the first insights into the potential role of iopofosine therapy in patients with heavily treated RRMM. In this population, AEs were predictable, and manageable with adequate supportive care. Based on the safety profile and evidence of biologic response, iopofosine is a novel option for patients with RRMM, warranting further clinical development.

## Figures and Tables

**Table 3:** Adverse events by dose level.

	Dose Cohort (mCi/m^2^)							
	A (n = 5)	B (n = 5)	C (n = 4)	D (n = 4)	E (n = 4)	F (n = 5)	G (n = 4)	Overall (n = 31)
AE (number of patients) *	5	5	4	4	4	5	4	31
≥Grade 3 AE (number of patients)	5	5	4	4	4	5	4	31
SAE (number of patients)	0	2	1	2	3	3	2	13
DLT (number of patients) ^†^	0	0	1	2	1	1	1	6
≥Grade 3 TEAE, by CTCAE Term								
Thrombocytopenia	1	3	4	4	4	4	3	23
Decreased lymphocyte count	5	5	4	4	4	1	0	23
Neutropenia	0	3	4	4	3	2	1	17
Anemia	1	2	2	4	1	4	2	16
Decreased WBC	1	4	3	4	3	0	0	15
Fatigue	0	0	1	2	0	0	1	4
Hypophosphatemia	1	0	1	0	1	1	0	4
Febrile neutropenia ^‡^	0	0	0	1	0	1	1	3
Pain/back pain/muscle spasm	0	0	0	1	1	0	1	3
Sepsis	0	1	0	0	1	0	0	2
Hyperglycemia	1	0	1	0	0	0	0	2
Hyponatremia	0	1	0	1	0	0	0	2
UTI	0	0	1	0	0	1	0	2
Dyspnea	0	0	0	1	1	0	0	2
Respiratory failure	0	1	0	0	0	1	0	2

Abbreviations: AE = adverse event; DLT = dose-limiting toxicity; SAE = serious adverse event; TEAE = treatment-emergent AE; UTI = urinary tract infection; WBC = white blood cell. * Determined to be related to, probably related to, or possibly related to iopofosine. ^†^ The data-monitoring committee reviewed all cases that met the definition of DLT. ^‡^ All cases of febrile neutropenia resolved without sequelae.

**Table 4:** Magnitude of early and late hematologic response.

	<60 mCi (n = 20) Mean (% Change)	≥60 mCi (n = 11) Mean (% Change)	Overall (n = 31) Mean (% Change)
Platelet count (×10^3^/mL)			
Baseline	205.7	173.6	194.3
Day 15	183 (−11)	167.1 (−3.7)	177.6 (−9)
Day 85	116.6 (−43)	101.4 (−42)	110.7 (−43)
WBC count (×10^3^/mL)			
Baseline	6.56	5.26	6.10
Day 15	5.47 (−17)	5.53 (+5)	5.49 (−10)
Day 85	4.74 (−28)	4.61 (−12)	4.72 (−23)
ANC (×10^3^/mL)			
Baseline	4.88	3.75	4.48
Day 15	4.77 (−2)	4.80 (+28)	4.78 (+7)
Day 85	3.61 (−26)	3.19 (−15)	3.46 (−23)
Lymphocytes (×10^3^/mL)			
Baseline	0.94	0.93	0.94
Day 15	0.30 (−68)	0.35 (−62)	0.32 (−66)
Day 85	0.60 (−36)	0.67 (−28)	0.63 (−33)
RBC count (×10^6^/mL)			
Baseline	3.57	3.88	3.68
Day 15	3.47 (−3)	3.90 (+1)	3.62 (−2)
Day 85	3.11 (−13)	3.07 (−21)	3.09 (−16)
Hemoglobin (g/dL)			
Baseline	11.38	12.15	11.65
Day 15	11.17 (−2)	12.31 (+2)	11.57 (−1)
Day 85	10.13 (−11)	9.96 (−18)	10.1 (−13)

Abbreviations: ANC = absolute neutrophil count; RBC = red blood cell; WBC = white blood cell.

## Data Availability

The data presented in this study are available on request from the corresponding author due to privacy concerns.

## Abbreviations

The following abbreviations are used in this manuscript:

^131^I	Iodide 131
AE	Adverse event
ANC	Absolute neutrophil count
ASCT	Autologous stem cell transplant
BCMA	B-cell maturation antigen
BSA	Body surface area
CAR-T	Chimeric antigen receptor, T-cell
CBR	Clinical benefit rate
CI	Confidence interval
DEX	Dexamethasone
DLT	Dose-limiting toxicity
DMC	Data-monitoring committee
DOR	Duration of response
ECOG	Eastern Cooperative Oncology Group
eGFR	Estimated glomerular filtration rate
FISH	Fluorescence in situ hybridization
FLC	Free light chain
IMWG	International Myeloma Working Group
IV	Intravenously
M	Monoclonal
MM	Multiple myeloma
MoA	Mechanism of action
NE	Not estimable
ORR	Overall response rate
PFS	Progression-free survival
PR	Partial response
PS	Performance status
RBC	Red blood cell
RRMM	Relapsed/refractory multiple myeloma
SAE	Serious adverse event
SD	Standard deviation
SD	Stable disease
TEAE	Treatment-emergent adverse event
UTI	Urinary tract infection
VDT-PACE	Bortezomib, dexamethasone, thalidomide, cisplatin, doxorubicin, cyclophosphamide, and etoposide
WBC	White blood cell
